# Efficacy and safety of letermovir prophylaxis for cytomegalovirus infection after hematopoietic stem cell transplantation

**DOI:** 10.1097/BS9.0000000000000178

**Published:** 2024-01-10

**Authors:** Wen-Wen Li, Yong-Mei Zhang, Meng-Zhu Shen, Xiao-Dong Mo

**Affiliations:** a Peking University People’s Hospital, Peking University Institute of Hematology, National Clinical Research Center for Hematologic Disease, Beijing Key Laboratory of Hematopoietic Stem Cell Transplantation, Beijing, China; b Department of Hematology, Qingdao Women and Children’s Hospital, Qingdao, China; c Department of Hematology, Shijiazhuang People’s Hospital, Shijiazhuang, China; d Research Unit of Key Technique for Diagnosis and Treatments of Hematologic Malignancies (2019RU029), Chinese Academy of Medical Sciences, Beijing, China

**Keywords:** Allogeneic hematopoietic stem cell transplantation, Cytomegalovirus, Letermovir, Prophylaxis

## Abstract

Letermovir is a specific inhibitor of cytomegalovirus (CMV) terminase complex. Several studies have reported that letermovir can effectively prevent CMV activation after allogeneic hematopoietic stem cell transplantation (allo-HSCT). We aimed to identify the efficacy and safety of letermovir prophylaxis for CMV infection after allo-HSCT with a systemic review and meta-analysis. A literature search was conducted following the Preferred Reporting Items for Systematic Reviews and Meta-analyses statement. PubMed and Embase databases were searched. A total of 28 studies were included. The incidence of CMV activation at 14 weeks after HSCT was 0.10 (95% confidence interval [CI], 0.06–0.18), which was 0.10 (95% CI, 0.04–0.21) and 0% in adult and children (2 studies were included and both of them were 0%). In addition, the incidence of CMV activation at 14 weeks after allo-HSCT was 0.11 (95% CI, 0.06–0.21) and 0.07 (only 1 study included), respectively, in retrospective and prospective studies. The incidence of CMV activation at 100 and 200 days after HSCT was 0.23 (95% CI, 0.16–0.33) and 0.49 (95% CI, 0.32–0.67), respectively. The incidence of CMV disease at 14 weeks and at 6 months after HSCT was 0.01 (95% CI, 0.01–0.02) and 0.03 (95% CI, 0.01–0.09), respectively. Thus, our systemic review and meta-analysis suggested that letermovir prophylaxis was safe and effective for CMV activation after allo-HSCT.

## 1. INTRODUCTION

Allogeneic hematopoietic stem cell transplantation (allo-HSCT) is one of the most important treatments for patients with hematological malignancies and non-malignant hematologic disorder.^1^ Infections are the most common and significant cause of mortality and morbidity after allo-HSCT.^2^ Viruses contribute to nearly one-third of infection-related mortality,^3^ and cytomegalovirus (CMV) is the most common viral infection after allo-HSCT. The 1-year cumulative incidence of CMV activation was 55.0% after allo-HSCT, which is 23.5% to 48%,^4–6^ 42% to 66%,^7–9^ and 54% to 87%,^10–12^ respectively, in identical sibling donor (ISD), haploidentical-related donor (HID), and unrelated donor (URD) HSCT recipients. In addition, nearly 50% of these patients may experience refractory/recurrent CMV infection which significantly increases the risk of CMV disease and non-relapse mortality (NRM) after allo-HSCT.^13^

Although most of the allo-HSCT recipients receive acyclovir for herpes simplex virus prevention, it cannot prevent CMV activation. There are several anti-CMV agents, such as ganciclovir, foscarnet, and cidofovir; however, most of them are not suitable for CMV prophylaxis because of their toxicities. For example, ganciclovir can cause severe myelosuppression, and foscarnet and cidofovir can cause severe or even irreversible nephrotoxicity.^14–17^ Thus, how to prevent CMV activation safely and effectively is important to improve the clinical outcomes of allo-HSCT recipients.

Letermovir is a 3,4-dihydro-quinazoline-4-yl-acetic acid derivative that inhibits viral terminase complex inhibitor^18,19^ Several studies observe that letermovir can prevent CMV activation after allo-HSCT.^20–22^ However, most of them were retrospective studies and the efficacy of CMV prophylaxis was inconsistent among these studies.

Thus, we aimed to further identify the efficacy and safety of letermovir prophylaxis for CMV infection after allo-HSCT through a systemic review and meta-analysis.

## 2. METHODS

### 2.1. Inclusion criteria

The inclusion criteria were as follows: patients of any race, any sex, and all ages; those diagnosed with CMV infection after allo-HSCT; and those using letermovir for CMV infection after allo-HSCT. Reviews, case reports, duplicates, and conference abstracts were excluded.

### 2.2. Search strategy

A literature search was conducted following the Preferred Reporting Items for Systematic Reviews and Meta-analyses statement.^23^ The PubMed and Embase databases were searched, published from January 2017 to December 2022, with the search strategy following the Population (allo-HSCT recipients), Intervention (letermovir for prevention of CMV infection), Outcomes (CMV infection, CMV disease, adverse events, overall survival [OS]), and Study framework (retrospective, prospective non-randomized, and randomized trials).^24^

### 2.3. Data extraction and outcomes

Information on the following was extracted: study characteristics (eg, study framework, first author, publish year), patients (eg, age, number, and diagnosis), and outcome parameters during the follow-up period. CMV infection at 14 weeks after allo-HSCT was chosen as the primary end point. CMV infection at other time points (ie, 100 days, 6 months, 200 days, and at any time) after HSCT, CMV disease after allo-HSCT, adverse event, and OS were chosen as secondary end points. Missing data were documented as “not available (NA).” All data were extracted according to the Cochrane Handbook for Systematic Reviews of Interventions.^25^

### 2.4. Statistical analysis

The “meta” package version 4.16-2^26^ was used to perform the meta-analysis (R Project for Statistical Computing, version 4.0.5). Statistical heterogeneity among studies was assessed using the *I*^2^ statistics and Cochran *Q*-test. The random effects model was adopted, with the heterogeneity test showing *I*^2^ > 50% and *P* < .10. The subgroup comparison of adults and children was also conducted. The null hypothesis was set to no difference. A *P* value <.05 was considered statistically significant to reject the null hypothesis. The results were analyzed by the boxplot using “ggplot2” package version 3.3.5.^27^

## 3. RESULTS

### 3.1. Included studies

**Figure 1.**
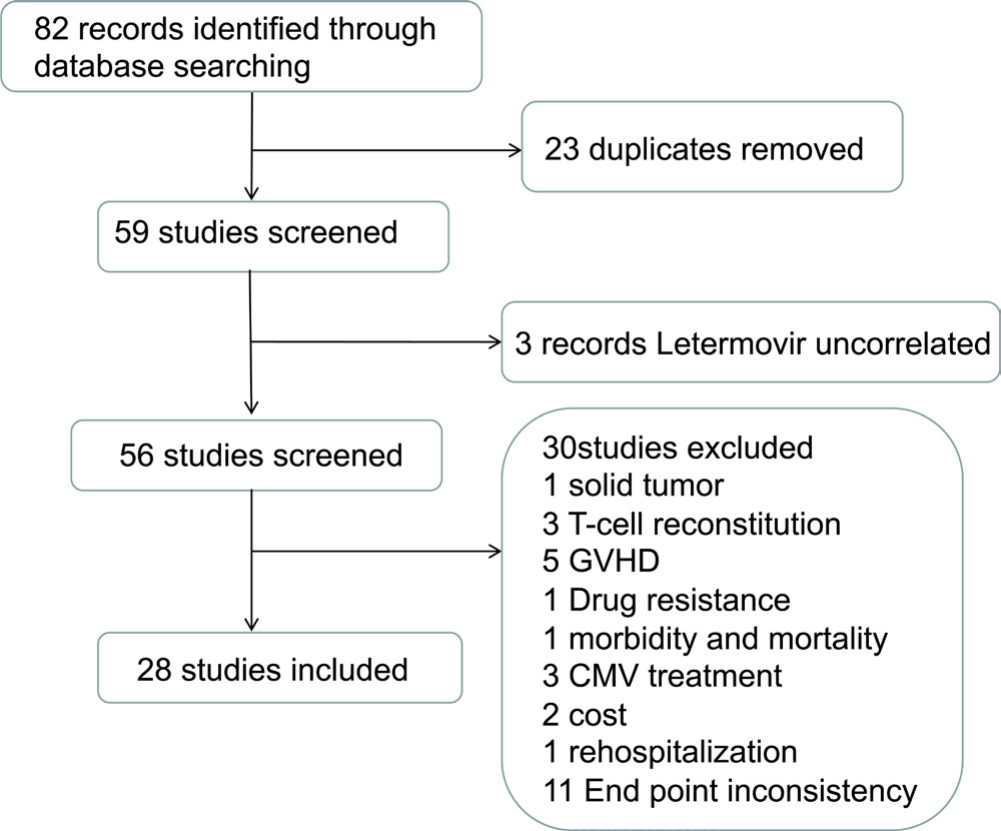
Selection scheme of studies. CMV = cytomegalovirus, GVHD = graft-versus-host disease.

A total of 28 studies with 2389 patients were included in this meta-analysis (Tables 1 and 2, **Fig. 1**; Supplementary Table 1, http://links.lww.com/BS/A76).

**Table 1:** Main characteristics of 28 included studies.

Studies	Study design	N	CMV infection					CMV disease			
			At 100 d	At 14 wk	At 6 mo	At 200 d	At any time	At 14 wk	At 6 mo	At 200 d	At any time
Marty et al 2017^32^	Prospective	373	NA	25	57	NA	NA	1	5	NA	NA
Lin et al 2019^48^	Retrospective	53	NA	NA	NA	NA	2	NA	NA	NA	NA
Malagola et al 2020^30^	Retrospective	60	NA	6	11	NA	NA	1	1	NA	NA
Anderson et al 2020^46^	Retrospective	25	NA	NA	NA	10	NA	NA	NA	0	0
Johnsrud et al 2020^37^	Retrospective	114	49	NA	NA	NA	NA	NA	NA	NA	NA
Sharma et al 2020^41^	Retrospective	32	7	NA	NA	NA	NA	NA	NA	NA	NA
Chen et al 2021^42^	Retrospective	60	NA	NA	12	NA	1	NA	NA	NA	NA
Mori et al 2021^43^	Retrospective	114	NA	NA	47	NA	NA	NA	NA	NA	NA
Royston et al 2021^44^	Retrospective	26	NA	NA	9	NA	NA	NA	NA	NA	NA
Wolfe et al. 2021^47^	Retrospective	119	NA	NA	NA	76	NA	NA	NA	NA	NA
Martino et al 2021^31^	Retrospective	204	NA	20	61	NA	NA	5	7	NA	NA
Derigs et al 2021^35^	Retrospective	80	11	NA	NA	NA	NA	NA	NA	NA	NA
Cassaniti et al 2021^28^	Retrospective	77	NA	26	NA	NA	NA	NA	NA	NA	NA
Serio et al 2021^40^	Retrospective	13	1	NA	NA	NA	NA	NA	NA	NA	NA
Sassine et al 2021^39^	Retrospective	123	19	NA	NA	NA	21	NA	NA	NA	NA
Hiraishi et al 2021^29^	Retrospective	460	NA	79	140	NA	NA	6	11	NA	NA
Beauvais et al 2022^20^	Retrospective	96	NA	15	NA	NA	NA	0	NA	NA	NA
Politikos et al 2022^33^	Retrospective	28	NA	0	NA	NA	NA	NA	NA	NA	NA
Gabanti et al 2022^36^	Retrospective	30	10	NA	NA	NA	NA	NA	NA	NA	NA
Daukshus et al 2022^49^	Retrospective	10	NA	NA	NA	NA	2	NA	NA	NA	NA
Richert-Przygonska et al 2022^34^	Retrospective	13	NA	0	NA	NA	NA	0	NA	NA	NA
Cheng et al. 2022^21^	Retrospective	4	NA	0	NA	NA	NA	NA	NA	NA	NA
Freyer et al 2022^22^	Retrospective	19	NA	NA	NA	4	NA	NA	NA	NA	NA
Yoshimura et al 2022^45^	Retrospective	38	NA	NA	1	NA	NA	NA	1	NA	NA
Mizuno et al 2022^38^	Retrospective	43	13	NA	NA	28	NA	NA	NA	NA	NA
Łojko et al 2022^52^	Retrospective	53	NA	NA	NA	NA	NA	NA	15	NA	NA
Robin et al 2020^50^	Prospective	80	NA	NA	NA	NA	4	NA	NA	NA	3
Studer et al 2020^51^	Retrospective	42	NA	NA	NA	NA	5	NA	NA	NA	NA

CMV = cytomegalovirus, NA = not available.

**Table 2:** Other characteristics of 28 included studies.

Studies	Median age/year (range)	HLA matching (n)				Diagnosis (n)										
		MRD	mMRD	MUD	mMUD	AML	ALL	AL	MDS	CLL	CML	MM	Lymphoma	AA/SAA/VSAA	MPN	Other
Marty et al 2017^32^	53 (18–75)	121	63	138	51	142	35	NA	63	NA	NA	NA	47	NA	NA	86
Lin et al 2019 (group 1)^48^	59 (20–74)	9	2	16	3	12	NA	3	13	NA	NA	2	5	NA	NA	4
Lin et al 2019 (group 2)^48^	54 (28–72)	2	0	8	1	4	NA	1	2	NA	NA	3	2	NA	NA	2
Malagola et al 2020^30^	52 (21–71)	11	NA	32	NA	NA	NA	32	NA	NA	NA	8	7	2	NA	9
Anderson et al 2020^46^	60 (NA)	0	NA	4	14	NA	NA	12	NA	NA	NA	NA	3	NA	4	3
Johnsrud et al 2020^37^	55.5 (22–77)	29	NA	NA	67	43	15	NA	28	NA	2	NA	14	7	NA	5
Sharma et al 2020^41^	50 (22–74)	NA	NA	NA	NA	15	10	NA	3	0	0	NA	1	NA	2	1
Chen et al 2021^42^	61 (19–73)	4	NA	16	10	17	7	NA	9	NA	NA	NA	9	NA	5	13
Mori et al 2021^43^	57 (15–75)	21	37	22	34	52	20	NA	NA	NA	NA	2	34	2	2	2
Royston et al 2021^44^	55.8 (NA)	4	NA	15	NA	18	8	NA	NA	NA	NA	NA	NA	NA	NA	NA
Wolfe et al 2021^47^	56 (21–74)	24	NA	62	9	44	20	NA	23	6	2	5	12	NA	NA	7
Martino et al 2021^31^	52 (18–75)	66	68	56	14	109	28	NA	19	NA	NA	NA	15	NA	NA	33
Derigs et al 2021^35^	58.5 (18–75)	21	1	45	10	31	NA	NA	23	NA	NA	NA	15	NA	NA	11
Cassaniti et al 2021^28^	58 (48–64)	11	NA	NA	41	NA	NA	47	16	NA	NA	2	6	NA	NA	4
Serio et al 2021^40^	43 (22–71)	NA	NA	NA	NA	9	2	NA	1	NA	NA	1	0	NA	NA	NA
Sassine et al 2021^39^	57 (18–93)	37	NA	58	NA	52	16	NA	14	NA	NA	NA	NA	NA	10	31
Hiraishi et al 2021^29^	53 (4–73)	164	295	NA	236	186	73	NA	63	NA	NA	NA	65	NA	NA	73
Beauvais et al 2022^20^	56 (NA)	NA	56	NA	NA	45	16	NA	9	1	1	1	12	0	4	7
Politikos et al 2022^33^	47 (26–65)	NA	NA	NA	NA	13	7	21	5	NA	NA	NA	2	NA	NA	0
Gabanti et al 2022^36^	59 (45–62)	NA	NA	NA	NA	13	3	NA	6	NA	0	2	0	NA	2	4
Daukshus et al 2022^49^	15.2 (10–17.6)	2	NA	3	5	3	5	NA	NA	NA	1	NA	NA	1	NA	NA
Richert-Przygonska et al 2022^34^	13.2 (7.1–16.9)	NA	NA	8	NA	NA	NA	8	NA	NA	NA	NA	2	3	NA	0
Cheng et al 2022^21^	16.1 (9.2–17.8)	NA	NA	1	1	1	2	NA	NA	NA	NA	NA	0	1	NA	NA
Freyer et al 2022^22^	64 (37–74)	NA	NA	NA	NA	9	NA	NA	NA	NA	NA	NA	NA	NA	NA	10
Yoshimura et al 2022^45^	49 (18–68)	5	NA	18	8	15	13	NA	4	NA	1	NA	2	NA	NA	3
Mizuno et al 2022^38^	52 (18–65)	6	0	16	2	16	8	NA	7	NA	NA	NA	6	NA	NA	6
Łojko et al 2022^52^	38 (5–70)	NA	NA	NA	NA	20	12	NA	4	NA	NA	NA	7	2	2	4
Robin et al 2020^50^	57 (19–72)	NA	NA	NA	NA	31	13	NA	16	NA	1	NA	NA	NA	4	10
Studer et al 2020^51^	43 (22–65)	NA	NA	NA	NA	14	4	NA	7	NA	3	NA	11	NA	NA	3

AA = aplastic anemia, AL = acute leukemia, ALL = acute lymphoblastic leukemia, AML = acute myeloid leukemia, CLL = chronic lymphocytic leukemia, CML = chronic myelogenous leukemia, HLA = human leukocyte antigen, MDS = myelodysplastic syndromes, mMRD = mismatched related donor, mMUD = mismatched unrelated donor, MM = multiple myeloma, MPN = myeloproliferative neoplasms, MRD = matched related donor, MUD = matched unrelated donor, NA = not available, SAA = severe aplastic anemia, VSAA = very severe aplastic anemia.

### 3.2. CMV infection after allo-HSCT

**Figure 2.**
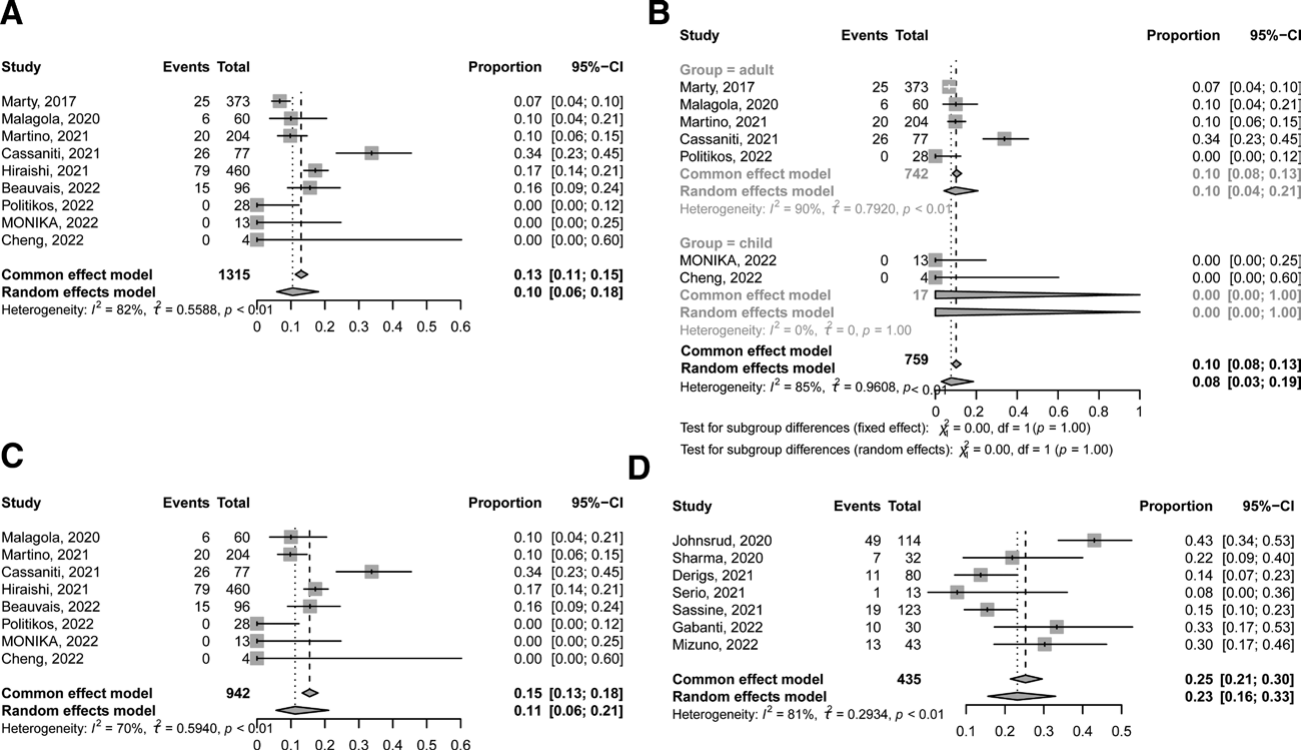
The incidence of CMV activation (A) at 14 wk after allo-HSCT in all the studies; (B) at 14 wk after allo-HSCT in adults and children; (C) at 14 wk after allo-HSCT in retrospective studies; (D) at 100 d after allo-HSCT in adults. allo-HSCT = allogeneic hematopoietic stem cell transplantation, CI = confidence interval, CMV = cytomegalovirus.

Nine studies^20,21,28–34^ including 1315 patients reported CMV activation at 14 weeks after allo-HSCT. The incidence of CMV activation at 14 weeks after HSCT was 10% (95% confidence interval [CI], 6%–18%) (**Fig. 2A**). In the subgroup analysis, 5 and 2 studies, respectively, reported the CMV activation at 14 weeks after allo-HSCT in adults and children. The incidence of CMV activation at 14 weeks after allo-HSCT in adults was 10% (95% CI, 4%–21%), which was comparable with that in children (0.0%, **Fig. 2B**). In addition, one of them was prospective study and the others were retrospective studies. In retrospective studies, the incidence of CMV activation at 14 weeks after allo-HSCT was 11% (95% CI, 6%–21%, **Fig. 2C**), which was 7% in prospective study.

Seven studies^35–41^ including 435 patients reported the CMV activation at 100 days after allo-HSCT. Only adults were enrolled in this analysis and the incidence of CMV activation at 100 days after allo-HSCT was 23% (95% CI, 16%–33%, **Fig. 2D**).

**Figure 3.**
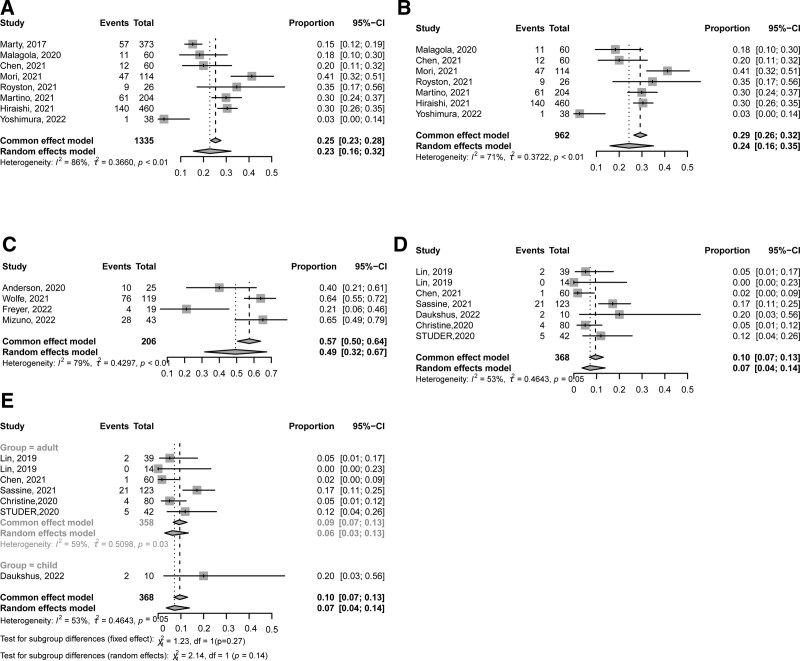
The incidence of CMV activation (A) at 6 mo after allo-HSCT in all the studies; (B) at 6 mo after allo-HSCT in retrospective studies; (C) at 200 d after allo-HSCT; (D) at any time after allo-HSCT; (E) at any time after allo-HSCT in adults and children. allo-HSCT = allogeneic hematopoietic stem cell transplantation, CI = confidence interval, CMV = cytomegalovirus.

Eight studies^29–32,42–45^ including 1335 patients reported the CMV activation at 6 months after allo-HSCT. The incidence of CMV activation at 6 months after allo-HSCT was 23% (95% CI, 16%–32%, **Fig. 3A**). One of them was prospective study and the others were retrospective studies. The incidence of CMV activation at 6 months after allo-HSCT was 24% (95% CI, 16%–35%, **Fig. 3B**) and 15%, respectively, for retrospective studies and the prospective study.

Four retrospective studies^22,38,46,47^ including 206 patients reported the CMV activation at 200 days after HSCT. The incidence of CMV activation at 200 days after allo-HSCT was 49% (95% CI, 32%–67%, **Fig. 3C**).

Six studies^39,42,48–51^ including 368 patients reported the CMV activation at any time after allo-HSCT. The incidence of CMV activation at any time was 7% (95% CI, 4%–14%, **Fig. 3D**). Five and 1 studies,^52^ respectively, were included for the analysis of CMV infection at any time after HSCT in adults and children, and the incidence of CMV infection at any time was 6% (95% CI, 3%–13%) in adults, which was comparable with that in children (20%, **Fig. 3E**).

### 3.3. CMV disease

**Figure 4.**
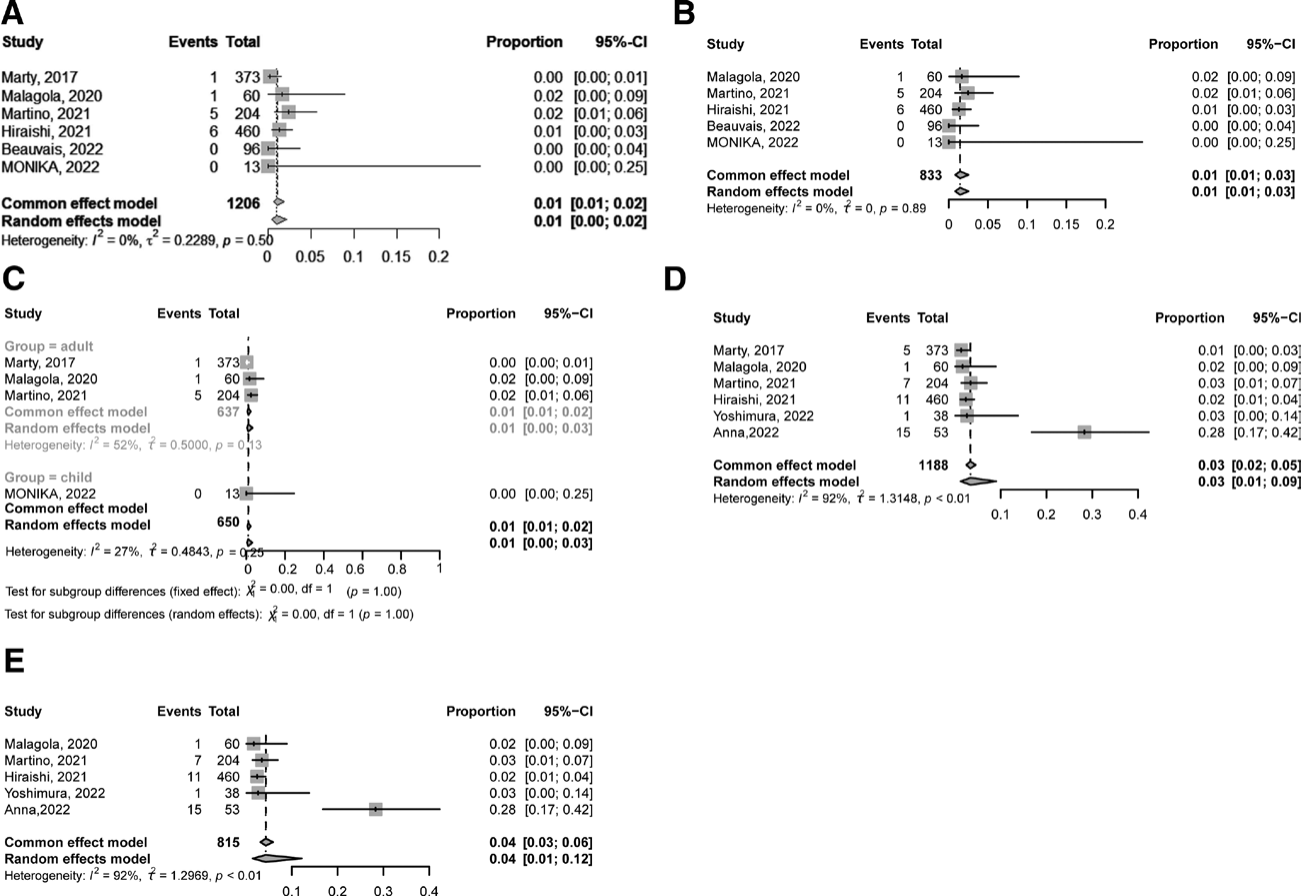
The incidence of CMV disease (A) at 14 wk after allo-HSCT; (B) at 14 wk after allo-HSCT in retrospective studies; (C) at 14 wk after allo-HSCT in adults and children; (D) at 6 mo after allo-HSCT; (E) at 6 mo after allo-HSCT in retrospective studies. allo-HSCT = allogeneic hematopoietic stem cell transplantation, CI = confidence interval, CMV = cytomegalovirus.

Six studies^20,29–32,34^ including 1206 patients reported the incidence of CMV disease at 14 weeks after allo-HSCT. The incidence of CMV disease at 14 weeks after allo-HSCT was 1% (95% CI, 1%–2%) (**Fig. 4A**). In retrospective studies, the incidence of CMV disease at 14 weeks after allo-HSCT was 1% (95% CI, 1%–3%, **Fig. 4B**). One prospective study was included in this analysis^32^ and the incidence was 0.3%. In addition, 3 and 1 studies, respectively, were included for the analysis of CMV disease at 14 weeks after allo-HSCT in adults and children. The incidence of CMV disease at 14 weeks after allo-HSCT was comparable between adults (1%, 95% CI, 0%–3%) and children (0%, **Fig. 4C**).

Six studies including 1188 patients reported the incidence of CMV disease at 6 months after allo-HSCT.^29–32,45,52^ The incidence of CMV disease at 6 months after allo-HSCT was 3% (95% CI, 1%–9%, **Fig. 4D**). In the 5 retrospective studies, the incidence of CMV disease at 6 months after allo-HSCT was 4% (95% CI, 1%–12%) (**Fig. 4E**). One prospective study was included,^32^ and the incidence of CMV disease at 6 months after allo-HSCT was 1%.

Only 1 retrospective study including 25 patients reported the incidence of CMV disease at 200 days after allo-HSCT.^46^ The incidence of CMV disease at 200 days after allo-HSCT was 0%.

Only 1 prospective study^50^ including 80 patients reported the incidence of CMV disease at any time after allo-HSCT. The incidence of CMV disease at any time after allo-HSCT was 4%.

### 3.4. Adverse events

Six studies reported the adverse events after letermovir prophylaxis (Supplementary Table 2, http://links.lww.com/BS/A77), and 4, 3, 3, 2, 2, 2, 2, 1, 2, 1, 2, 1, 1, 3, 1, 1, 1, and 2 studies showed the occurrence of graft-versus-host disease (GVHD), diarrhea, nausea, fever, rash, vomiting, cough, peripheral edema, fatigue, mucosal inflammation, headache, abdominal pain, ascites, acute kidney injury, hepatic function abnormal, decreased appetite, hypertension, and constipation after treatment (Table 3). Most studies^32,34,40,45^ show that no myelotoxicity of letermovir was found, which is particularly important in the context of the toxicity of other anti-CMV drugs.^52^

**Table 3:** The incidence of adverse events.

Adverse events	Incidence (cases per person)
Graft-versus-host disease	0.24–0.53
Diarrhea	0.01–0.75
Nausea	0.02–1.25
Fever	0.21–1.25
Rash	0.20–1.25
Vomiting	0.18–0.75
Cough	0.14–0.75
Peripheral edema	0.14
Fatigue	0.13–1.75
Mucosal inflammation	0.12
Headache	0.00–0.14
Abdominal pain	0.12
Ascites	0.01
Acute kidney injury	0.02–0.10
Hepatic function abnormal	0.01
Decreased appetite	0.10
Hypertension	0.08
Constipation	0.07–1.00

### 3.5. Overall survival

Two studies were included in the analysis of OS.^35,43^ The probability of OS at 6 months and at 1 year after allo-HSCT was 80.4% and 84%, respectively.

## 4. DISCUSSION

We observed that the incidence of CMV activation at 14 weeks and at any time was 10% and 7%, respectively. In addition, the incidence of CMV disease at 14 weeks and at any time was 1% and 4%, respectively. This is the first systematic review and meta-analysis identifying the efficacy and safety of letermovir prophylaxis for CMV activation after allo-HSCT.

Drugs currently used for CMV treatment, such as ganciclovir, and foscarnet, cannot be routinely used for CMV prophylaxis because of myelosuppression and nephrotoxicity. According to the published articles, the rate of acute kidney injury was only 2% to 10% cases per person and no myelosuppression event was observed after letermovir prophylaxis. These are the most important adverse events in CMV prophylaxis after allo-HSCT particularly for those receiving HID HSCT who have a higher risk of poor graft function and renal injury.^20,21,29,32,39,41^ In addition, most side effect of letermovir is mild which suggested that letermovir is suitable for CMV prophylaxis after allo-HSCT.

CMV disease was one of the most important risk factors for NRM, and the incidence was 23.5% to 48%, 54% to 87%, and 42% to 66%, respectively, for those receiving ISD,^4–6^ URD,^10–12^ and HID HSCT.^7–9^ We observed that the incidence of CMV disease was only 4% at any time after allo-HSCT, which was significantly decreased by letermovir prophylaxis.

CMV reactivation rate at 14 weeks after HSCT was only 10% after letermovir prophylaxis for CMV activation after allo-HSCT, which suggested that letermovir could effectively prevent CMV activation within 3 months after allo-HSCT. However, we observed that the incidence of CMV activation increasing beyond 3 months after allo-HSCT, and the incidence of CMV activation at 200 days after HSCT could achieve as high as 49%. Some authors reported that frequent delayed-onset CMV infections may be associated with letermovir discontinuation.^53^ In addition, late-clinically significant CMV infection may be correlated with HLA-mismatched donors or CMV-IgG–negative donors.^54^ However, some authors reported that letermovir may delay CMV-specific cellular reconstitution, possibly related to decreased CMV antigen exposure.^55^ Thus, how to prevent the late-onset CMV infection should be further identified.

However, there were some questions that could not be resolved by our study. Most of the published studies did not compare the clinical outcomes of letermovir prophylaxis among different donor types, and we could not further identify its efficacy and safety in particular allo-HSCT recipients (eg, HID HSCT recipients). In addition, the information about letermovir prophylaxis among different underlying disease, disease status, and comorbidities burden before HSCT was also rare. To draw a more significant conclusion, we listed odds ratios/hazard ratios in Supplementary Table 3, http://links.lww.com/BS/A78. Lastly, few post-engraftment variables were available in these articles and most of the studies were retrospective, which were the limitations of this paper.

## 5. CONCLUSION

In summary, our systemic review and meta-analysis suggested that letermovir prophylaxis was safe and effective for CMV activation after allo-HSCT.

## ACKNOWLEDGMENTS

National Key Research and Development Program of China (grant no. 2022YFC2502606), Tongzhou District Distinguished Young Scholars (grant no. JCQN2023009), the National Natural Science Foundation of China (grant nos. 82170208, 82200239), and CAMS Innovation Fund for Medical Sciences (grant nos. 2019-I2M-5-034, 2022-I2M-C&T-B-121). Thanks for the support from Yinzhu Jin in terms of data collection and analysis.

## Supplementary Material


