# Supplementary material for: Efficacy and safety of letermovir prophylaxis for cytomegalovirus infection after hematopoietic stem cell transplantation
Source: Blood Sci. 2024 Jan 10;6(1):e00178. doi: 10.1097/BS9.0000000000000178 (PMC10781138; doi:10.1097/BS9.0000000000000178)
Supplement: Supplementary file 1 [file bs9-6-e00178-s001.pdf]

Supplementary Table 1. Included Studies by Subgroup

Subgroup	Studies included, No. (%)
**CMV infection**	n=27
At 14 weeks	
Adult	5 (18.5)
Child	2 (7.4)
Retrospective	8 (29.6)
Prospective	1 (3.7)
At 100 days	
Adult	7 (25.9)
Child	0 (0)
At 6 months	
Retrospective	7 (25.9)
Prospective	1 (3.7)
At any time	
Adult	5 (18.5)
Child	1 (3.7)
**CMV disease**	n=10
At 14 weeks	
Adult	3 (30.0)
Child	1 (10.0)
Retrospective	5 (50.0)
Prospective	1 (10.0)
At 6 months	
Retrospective	5 (50.0)
Prospective	1 (10.0)
