# Supplementary material for: Efficacy and safety of letermovir prophylaxis for cytomegalovirus infection after hematopoietic stem cell transplantation
Source: Blood Sci. 2024 Jan 10;6(1):e00178. doi: 10.1097/BS9.0000000000000178 (PMC10781138; doi:10.1097/BS9.0000000000000178)
Supplement: Supplementary file 2 [file bs9-6-e00178-s002.pdf]

## Supplementary Table 2. Included Studies by Subgroup

| Subgroup | Studies included, No. (%) |
|---|---|
| Adverse event | n=6 |
| GVHD | 4 (66.7) |
| Diarrhea | 3 (50.0) |
| Nausea | 3 (50.0) |
| Fever | 2 (33.3) |
| Rash | 2 (33.3) |
| Vomiting | 2 (33.3) |
| Cough | 2 (33.3) |
| Peripheral edema | 1 (16.7) |
| Fatigue | 2 (33.3) |
| Mucosal inflammation | 1 (16.7) |
| Headache | 2 (33.3) |
| Abdominal pain | 1 (16.7) |
| Ascites | 1 (16.7) |
| Acute kidney injury | 3 (50.5) |
| Hepatic function abnormal | 1 (16.7) |
| Decreased appetite | 1 (16.7) |
| Hypertension | 1 (16.7) |
| Constipation | 2 (33.3) |

GVHD, graft versus host disease
