# Supplementary material for: Efficacy and safety of letermovir prophylaxis for cytomegalovirus infection after hematopoietic stem cell transplantation
Source: Blood Sci. 2024 Jan 10;6(1):e00178. doi: 10.1097/BS9.0000000000000178 (PMC10781138; doi:10.1097/BS9.0000000000000178)
Supplement: Supplementary file 3 [file bs9-6-e00178-s003.pdf]

Supplementary Table 3. Hazard Ratio/Odds Ratio provided from included studies

Study	Groups	Hazard Ratio/Odds Ratio
Freyer,2022	Letermovir vs. high-dose valacyclovir	By day 100: 0.1 (0.14–0.71)<br>By day 200: 0.14 (0.02–1.05)<br>By 1 year: 0.36 (0.14–0.94)
Mori,2021	Letermovir prophylaxis:<br>Yes vs. no	0.65 (0.44–0.97)
Sassine,2021	Letermovir primary prophylaxis:<br>Yes vs. no	Refractory or Resistant CMV: 0.15 (0.04-0.58);<br>Clinically significant CMV infection: 0.26 (0.16-0.41)
Wolfe,2021	Letermovir: Yes vs. no	Clinically significant CMV infection: 0.18 (0.10-0.32)
Derigs,2021	Letermovir prophylaxis:<br>Yes vs. no	Clinically significant cytomegalovirus infection by day 100: 0.29 (0.15-0.57)
